# Easy and Green Method to Fabricate Highly Thermally Conductive Poly(decamethylene terephthalamide)/Graphite Nanoplatelets Nanocomposite with Aligned Structure

**DOI:** 10.3390/molecules29133141

**Published:** 2024-07-02

**Authors:** Pengyuan Xu, Tianhao Ai, Pingli Wang, Junhui Ji

**Affiliations:** 1 National Engineering Research Center of Engineering Plastics and Ecological Plastics, Technical Institute of Physics and Chemistry, Chinese Academy of Sciences, Beijing 100190, China; xupengyuan19@mails.ucas.ac.cn (P.X.); jhji@mail.ipc.ac.cn (J.J.); 2 Hainan Degradable Plastics Technology Innovation Center, Haikou 571137, China

**Keywords:** thermal conductivity, composite material, ball milling, semi-aromatic polyamides

## Abstract

With the development of miniaturization and integration of electrical and electronic equipment, the heat accumulation problems caused by the long-term operation of devices have become more and more serious. High thermal-conductivity and high-performance plastic composites have attracted significant interest from both academia and industry. Numerous studies have been recently conducted to enhance the thermal conductivity (TC) of nanofiller-filled polymeric composites. However, the homogeneous dispersion and directional arrangement of nanofillers in the resin matrix are the key factors limiting their effectiveness in enhancing thermal conductivity. Based on the feasibility considerations of mass production and industrial application, this paper reports on a novel preparation method of Poly(decamethylene terephthalamide)/graphite nanoparticle (GNP) nanocomposites with high thermal conductivity. Without borrowing solvents or other reagents, this method can effectively strip the inexpensive scaled graphite into nanoscale for its uniform dispersion and orientation arrangement by relying only on mechanical external forces. The whole technology is simple, green, and easy to industrialize. The fillers were well-dispersed and aligned in the PA10T, which played a role in significantly enhancing the thermal conductivity of the PA10T. In addition, we found that the thermal conductivity of the composites reached 1.20 W/(m·K) at 10 wt% filler content, which was 330% higher than that of the pure matrix. The mechanical properties of the composites were also significantly improved. This work provides guidance for the easy fabrication of thermally conductive composites with aligned structures.

## 1. Introduction

As the miniaturization and integration of electronic devices evolves, undesired heat accumulation problems have become ever more serious in the long-term operation of devices. In order to address this problem, various new materials have been developed for heat management [1,2,3]. The capability to enhance and tune engineering properties gives polymer functional composites competitive advantages over neat polymer and other metallic materials. In addition, polymer composites also have the characteristics of low production cost, easy processability, and light weight; they have gradually become preferred materials in thermal management. As an important class of special engineering plastic matrices, PA10T is currently the only commercially biobased semi-aromatic polyamide and is also widely used in the abovementioned application fields [4,5,6]. PA10T has many unique advantages that cannot be replaced, including a high transition temperature (T_g_, above 119 °C), excellent thermal stability, superior mechanical properties, and low water absorption. Unfortunately, the poor thermal conductivities (0.2–0.4 W/(m·K)) of polyamides present a major bottleneck for further applications of the material [7,8]. Thus, in order to enhance the thermal conductivity, the introduction of inorganic thermally conductive additives to the PA10T matrix has come to be regarded as an effective method. At present, there are many thermal conductive fillers used for polymer composites, such as hexagonal boron nitride (h-BN) [9,10], silicon carbide (SiC) [11], aluminum oxide (Al_2_O_3_) [12], graphene [13], carbon nanotubes (CNTs) [14], and so on. However, limited by the high prices of most fillers (h-BN, SiC, graphene, CNTs etc.), the extensive application of thermal conductive polymer composites in industrial sectors is still far below expectations. Therefore, there is a great demand for the development of low-cost and highly thermal conductive special engineering polymer composites.

It is well known that flake graphite is a platelet morphology carbon allotrope that is abundantly available in nature. Due to the low cost, light weight, high intrinsic thermal conductivity, and low coefficient of thermal expansion, it endows composites with higher mechanical strength and electrical conductivity compared with those containing spherical graphite at the same filler content [15,16,17]. However, flake graphite is composed of microscale aggregations of stacked graphene layers; the amount of exfoliated graphite is low. The simple mixing of polymers with graphite is more likely to cause uneven dispersion of graphite and form large-scale defects, as the filler is distributed in the matrix at macroscale, which is not conducive to the improvement of composite properties and the formation of thermal conductive pathways. Generally, when the number of graphite layers are 10 or more, the material is referred to as graphite nanoplatelets (GNPs), and in the case of fewer than 10 layers, as graphene. Thus, it is important to study how to prepare graphene or graphite nanoplatelets from graphite, and to improve the dispersion of high loading fillers. At present, the common preparation methods for exfoliating graphite are chemical vapor deposition [18], liquid-phase exfoliation [19], the oxidation-reduction method [20] etc. Due to their high cost, complicated procedures and many influence factors, these methods are difficult for industrial-scale production.

Ball milling techniques are often used to exfoliate graphite for preparing graphene or graphite nanoplatelet functional composites. T.S. Ong [21] ball milled graphite under different atmospheres; the resulting samples were still crystalline with low specific surface areas but had large stacking faults and were highly anisotropic. Wei et al. [22] prepared PA6/graphene nanoplates by solid-state shear milling and also demonstrated the exfoliation process of graphite. The thermal conductivity of the composites, with 5.0 wt% of graphene loading, was increased by 85% and the yield strength was also appreciably enhanced. Roshr et al. [23] used atactic polypropylene as a dispersant to exfoliate graphite in toluene by the method of wet ball milling, the graphite was efficiently exfoliated and graphite nanosheets were implemented. The procedure produced single flakes of GN with a crystallite out-of-plane thickness as low as 17.7 nm. The above research showed that the ball milling method can effectively strip the graphite to obtain a finer-scale (or even nanoscale) dispersion, which has lain a foundation for the enhancement of the thermal conductivity of the composite material. However, the thermal conductivity of the composite material is not only related to the size of the thermal conductive fillers but also closely related to its dispersion and arrangement in the matrix, especially for systems of two-dimensional thermally conductive fillers such as graphene and carbon nanotubes [24,25,26]. It is difficult to construct an ideal interconnection network of thermal conduction paths using only the random distribution of thermally conductive fillers, which limits the development of high-level thermal conductivity values in the composites. In addition to improving thermal conductivity, the orientation arrangement of two-dimensional fillers in the composites tends to result in an effective enhancement of the mechanical properties of the materials. This structure enables the composites to withstand greater loads along the orientation direction during the tensile process, resulting in further reinforcement of the mechanical properties of the materials.

Hence, the scope of this work is to develop a simple, green, and highly industrializable method to prepare aligned graphite nanoplatelet-filled semi-aromatic polyamide 10T nanocomposites. First, in situ ball milling is used to mix the graphite and PA10T particles together. The ball milling exerts greater shear force to graphite, which can provide a better exfoliation effect and more uniform dispersion of the GNPs in the matrix. More importantly, the exfoliation process is completed in situ inside the PA10T matrix in a solid state, which effectively solves the problem of dispersing and preventing the restacking and re-agglomeration of graphite nanoplatelets in this process. Second, the mixtures of ball-milled composites are melt-blended by a twin-screw extruder; nanofiller alignment in the composites is achieved through stretching or shearing via melting extrusion. This method does not require prior exfoliation of graphite, or other chemical methods to prepare graphene beforehand; therefore, it has promising prospects in industrial applications. We also compare the mechanical properties and thermal conductivity of two kinds of composites filled with randomly distributed and aligned GNPs. Our research provides a novel idea for dispersing aligned GNPs in semi-aromatic polyamide without incorporating a compatibilizer, which makes the method more aligned with industrial-scale processes for producing highly thermally conductive composites with excellent mechanical properties.

## 2. Experimental

### 2.1. Materials

Bio-based semi-aromatic Polyamide 10T was synthesized by a one-pot polycondensation reaction (see Supporting Information). The intrinsic viscosity of PA10T was 0.95 dL/g. Flake graphite (100 meshes) was purchased from Qingdao Tianheda Graphite Co., Ltd., Qingdao, China. Detailed information on the reagents and preparation of PA10T are given in the Supplementary Materials.

### 2.2. Fabrication of PA10T/GNP Composites

**Figure 1:**
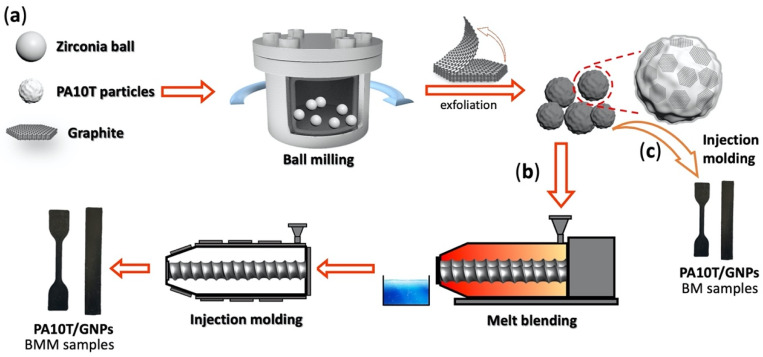
Schematic illustration of preparation procedures of PA10T/GNP nanocomposites: (**a**) ball milling; (**b**) BMM samples; and (**c**) BM samples.

The fabrication process of the PA10T/GNP composites is shown in Figure 1. First, PA10T particles (about 60 mesh, 1 kg) and graphite were mixed together, and the graphite content in the mixtures was controlled to be 2.5, 5.0, 7.5, and 10.0 wt%. The mixtures were added to a zirconia resonance ball milling machine (100 W, 47.8 Hz, GZM-5) and treated for 3 h. The ball milling process was conducted using a resonance ball milling in a 3 dm^3^ stainless steel jar with a zirconia ball of 7 mm in diameter, and a ball-to-power weight ratio of 6:1. In our preliminary experiment, ball milling for 3 h was effective in exfoliating graphite, and thus was chosen to study the effects of the content of graphite nanoplatelets on the structural changes and properties of PA10T/GNPs. Second, the mixtures, after ball milling, were melt-blended by a twin-screw extruder (Process 11 Extruder, Haake, Vreden, Germany) with a temperature of around 320 °C and a screw speed of 120 rpm. Prior to the injection molding, the compound was thoroughly dried in a vacuum oven at 90 °C for 12 h to remove water. Finally, the samples were molded with an injection molding machine to obtain specimens for measuring the mechanical properties and thermal conductivity. For comparison, PA10T/GNP nanocomposites were prepared by ball milling and injection molding, and are hereinafter labeled as BM samples in this paper. In addition, PA10T/GNPs were prepared by ball milling, melt-blending and injection molding, and are hereinafter labeled as BMM samples in this paper. It should be emphasized that in the ball milling process, ball milling was used to exfoliate graphite with the aim of dispersing the graphite nanoplatelets in the PA10T. In the melt-blending process, twin-screw extrusion further improved the dispersion of the fillers and induced alignment of the fillers in the matrix.

### 2.3. Characterization and Measurement

For the atomic force microscope study, the graphite nanoplatelets were separated from the compound powder according to the following process: the sample powder was dispersed in ethanol (0.01, *w*/*w*%) and sonicated for 5 min. Then, 4 μL of the upper part of the suspension was dropped on a silicon wafer. AFM was carried out using a Bruker (Billericam, MA, USA) Multimode-8. The graphite nanoplatelet images obtained were analyzed by NanoScope Analysis software version 1.40.

Raman Spectroscopy was carried out using a microscopic confocal laser-Raman spectrometer (Renishaw, Wotton-under-Edge, UK). Data were acquired using a laser power 10 mW at 532 nm.

Scanning electron microscopy (SEM) images were recorded on a HITACHI (Tokyo, Japan) S-4800 to analyze the distribution of GNPs in the matrix, and the samples were sputtered and coated with gold before observation by SEM.

X-ray micro-computed tomography (micro-CT) was performed on a SkyScan (Edinburgh, UK) 1272 Bruker for observing the morphology and dispersion of GNPs in the matrix. The X-ray source was operated at a voltage of 25 kv and a current of 193 μA. The dimensions of the samples were approximately 4 × 4 × 2 mm^3^.

The X-ray diffraction (XRD) patterns of the samples were recorded on a Bruker D8 focus instrument using CuKα radiation in the 2θ range from 2° to 60° at a scanning step of 0.2°/s.

The thermal conductivity was measured using Tci (Falls Church, VA, USA) C-THERM at 25 °C. Each composite sample with a dimension (Φ = 25 mm, h = 10 mm) was prepared by injection molding.

Differential scanning calorimetry (DSC) measurements were performed on a Mettler (Greifensee, Switzerland) DSC1 instrument at a heating and cooling rate of 10 K/min under a nitrogen atmosphere. The second heating curves were chosen to determine the melting temperature of the samples.

Thermal (Waltham, MA, USA) gravimetric analysis (TGA) was performed on a TA Q50 instrument at a heating rate of 10 °C/min from 25 °C to 600 °C under N_2_.

Tensile tests were performed on an Instron-5699 (Norwood, MA, USA) instrument at a stretching rate of 5 mm/min according to GB/T 1042.2-1992 [27]. Notched impact strength was measured with a Wuzhong Material Tester (Wuzhong, China) XJ-300A according to GB/T 1843.1-2008 [28]. For each sample, at least five specimens were tested, and the average values are reported.

## 3. Results and Discussion

### 3.1. Characterization of GNPs and Their Dispersion

Resonance ball milling techniques are processed in a dry grinding condition without using solvents, providing the comprehensive interactions of high-frequency resonance, impact, collision, shear and grinding between the samples and the milling medium. The graphite particles can be gradually exfoliated in situ by the mechanical force for breaking the relative weak interlayer force, resulting in an effective decrease in layer numbers and uniform dispersal in the PA10T matrix during the resonance ball milling process.

**Figure 2:**
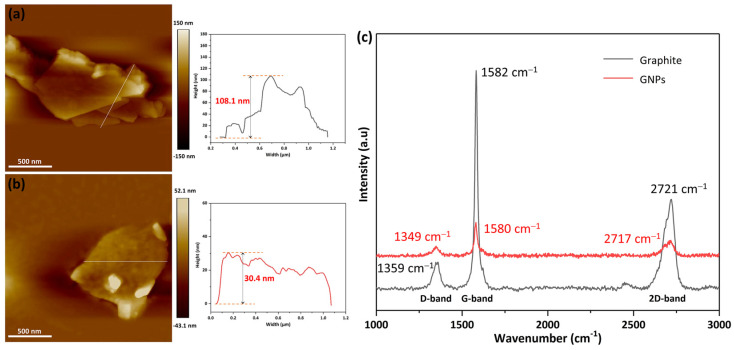
AFM images of: (**a**) flake graphite; (**b**) graphite nanoplatelets; and (**c**) Raman spectra of graphite, graphite nanoplatelets.

In order to detect the thickness of the graphite exfoliated by the resonance ball milling, the compounding powder was ultrasonically treated in ethanol and the graphite nanoplatelets were separated from the mixture. The AFM measurement of the flake graphite and GNPs are shown in Figure 2a,b, respectively. As presented in the AFM images, the flake graphite showed a multi-layer structure; the thickness of the graphite was generally about 108 nm g. The graphite became significantly thinner after ball milling for 3 h, and the average height of the graphite nanoplatelets was about 30.4 nm, as shown in Figure 2b. The Raman spectra of the flake graphite and GNPs are shown in Figure 2c. The peaks at around 1349, 1580 and 2717 cm^−1^ were attributed to D peak (defect mode owing to disruption of planar configuration), G peak (E_2g_ stretching mode of the graphitic crystalline structure), and 2D peak (signature of sp^2^ materials) of the carbon materials, respectively [29,30,31]. Compared with graphite, the sharp intensity decreased in D band and G band of graphite nanoplatelet inferred that the huge decline in graphite thickness and layer numbers. The above results demonstrated that ball milling could effectively exfoliate graphite and prepare graphite nanoplatelets in this work.

**Figure 3:**
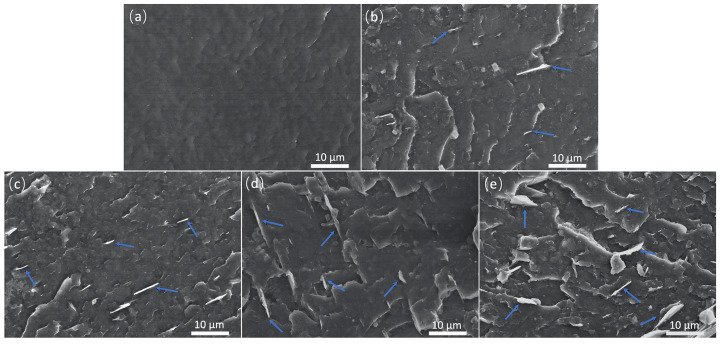
SEM images of cyro-fractured surfaces: (**a**) PA10T; (**b**) BMM-2.5 wt%; (**c**) BMM-5.0 wt%; (**d**) BMM-7.5 wt%; and (**e**) BMM-10.0 wt%.

The homogeneous dispersion of GNPs in the matrix is a key factor in fabricating high-performance nanocomposites [32]. The SEM images of the cryo-fractured surfaces of PA10T and PA10T/GNP (BMM-processed) nanocomposites are shown in Figure 3. The cryo-fractured surface of the neat PA10T is relatively smooth, showing a typical brittle fracture feature. The GNPs are well-dispersed and embedded in the PA10T, without obviously protruding from the surfaces in the matrix, as shown in Figure 3b–e [33]. However, when the GNP content reached 7.5 wt%, there were some large-sized graphite nanoplatelets on the surface, probably due to GNP self-agglomeration. Graphite nanoplatelets tend to aggregate on the surface of fractured surfaces, which visually led to a larger size of nanoplatelets, to a minor degree. In addition, it was observed that there were some bent graphite nanoplatelets and holes on the surfaces of composites at a relatively high GNP content, which is presumably due to the flexibility of graphite nanoplatelets and the space restriction between fillers, the stacking of some fillers was dislocated during the preparation of the composites. The SEM images of BMM samples demonstrate that the GNPs are uniformly dispersed in PA10T matrix, which could assist in significantly improving the comprehensive performance of composites, and will be discussed in detail in the subsequent parts. However, it was difficult to identify all the GNPs and observe the overall dispersion of the GNPs in the matrix due to the very similar gray color of the PA10T and GNPs.

**Figure 4:**
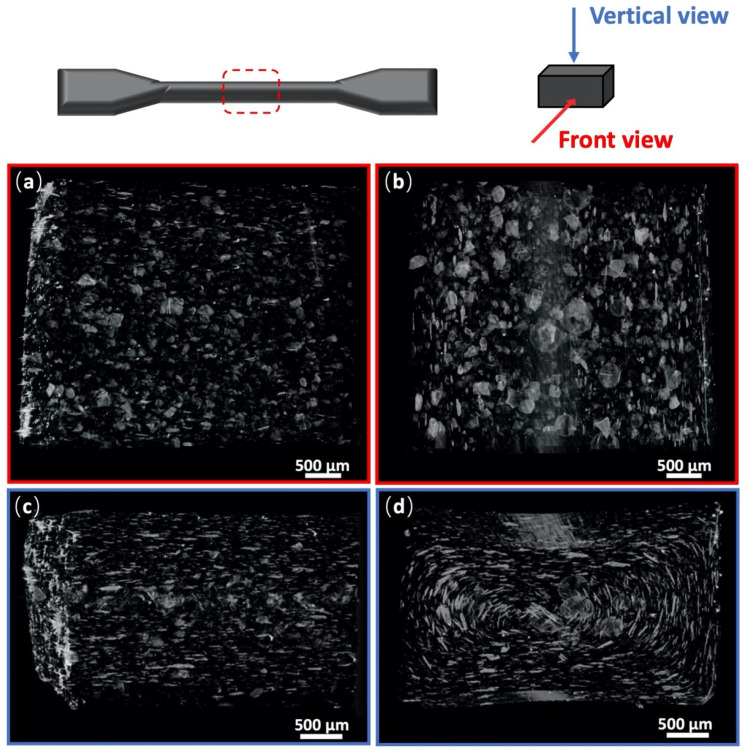
Micro X-ray CT images of: (**a**) front view and (**c**) vertical view of BMM-2.5 wt% GNPs; (**b**) front view; and (**d**) vertical view of BM-2.5 wt% GNPs.

To observe the overall dispersion of the GNPs in PA10T and compare the dispersion of GNPs prepared by the BMM and BM processes, micro-CT was further carried out to obtain a 3D vision of GNP dispersion in the matrix. Figure 4 presents the micro-CT images of PA10T/2.5 wt% GNPs (the BMM and BM samples) with dimensions of 4 × 4 × 2 mm^3^. As can be seen in Figure 4a, GNPs were uniformly dispersed in PA10T (the BMM-processed) without obvious aggregation, and the lateral sizes of most GNPs were between tens to a hundred micrometers. Compared with the imaging result of Figure 4b, obvious aggregations of GNPs were observed in the matrix fabricated by the BM process, thereby giving rise to the much bigger lateral sizes of the GNPs. The high shear forces and tension stresses during melt-blending and injection molding, and the flow of the PA10T viscous melt were able to break down some of these agglomerations when the BMM process was used. In addition, as can be seen in the vertical view of Figure 4c,d, the aligned GNPs formed in the BMM-processed platelets were preferentially aligned parallel to the flow direction of the polymer melt during melt-blending. The results show that the GNPs were well-dispersed and aligned in the PA10T matrix via the BMM process.

### 3.2. XRD Analysis

**Figure 5:**
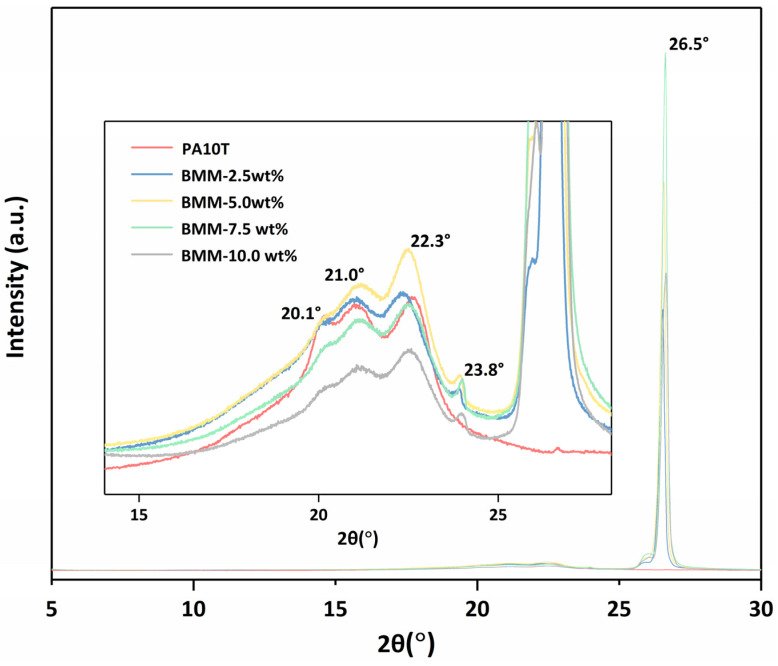
XRD patterns of PA10T and PA10T/GNP BMM samples.

XRD analysis was performed to evaluate the crystal structure of PA10T and PA10T/GNP BMM samples, as shown in Figure 5. A strong characteristic peak appeared at 2θ = 26.5° of the nanocomposites, attributed to the crystalline exfoliated from the GNPs during ball milling. A peak observed at 23.8° for the PA10T/GNP composites can be attributed to the randomly ordered GNPs with a corrugated structure. The characteristic graphite peak is more evident in patterns of composites with high GNP content [34,35]. PA10T exhibited three obvious diffraction peaks located at 2θ = 20.1°, 21.0° and 22.3°. The location of the above peaks for the samples of PA10T/GNP BMM compared to the neat PA10T [36] did not appear to change, which indicated that the successful introduction of the fillers had a minimal effect on PA10T crystal. However, the intensity of the diffraction peaks of the composites increased obviously when the GNPs were increased from 0.0 wt% to 5.0 wt%, which showed that the regularity of the PA10T molecular chain increased, probably due to the good dispersion and the nucleation effect of the GNPs. The GNPs functioned as the nucleation template, exhibiting a heterogeneous nucleation effect in the matrix because of its large specific surface area, which significantly improved the crystallinity of the nanocomposites, especially at a low GNP content. When the addition of GNPs continually increased, the intensity of the peaks significantly decreased and were lower than those of neat PA10T, which may have been caused by the rigid GNPs occupying the free volume in PA10T, thereby playing a pivotal part in heterogeneous nucleation for the matrix, but also strongly restricting the movement of the PA10T chain, leading to a reduction in crystallinity.

### 3.3. DSC and TGA Analysis

**Figure 6:**
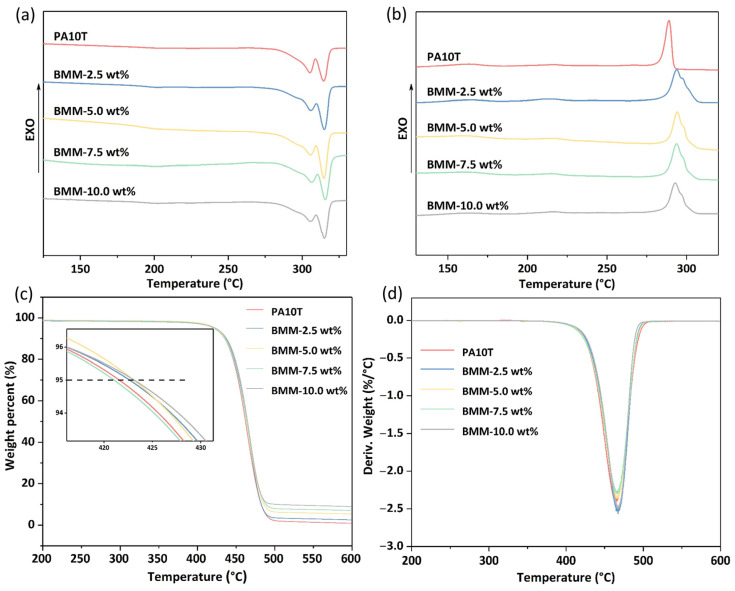
(**a**,**b**) DSC curves of the PA10T and PA10T/GNP BMM samples; (**c**) TGA; and (**d**) DTG curves of the PA10T and PA10T/GNP BMM samples.

The effect of GNPs on the melting–crystallization behavior and thermal stability of the PA10T/GNP nanocomposites were analyzed by DSC and TGA experiments, respectively. Figure 6a,b presents the DSC curves of the neat PA10T and PA10T/GNP nanocomposites BMM samples. Table 1 shows data from the DSC and TGA analyses of PA10T and PA10T/GNPs prepared via the BMM process. It can be seen that all the composites present distinct double-melting endothermic peaks, which resulted from the different crystal forms, the presence of different morphologies (lamellar thickness or perfection), and recrystallization during the subsequent heating. In our research, the reason for the double-melting behavior may have been due to the different morphologies of crystal, because the XRD results showed that the crystal form of PA10T did not change after the introduction of fillers. Moreover, the trace of the cooling curve presents only one crystallization peak for the PA10T/GNP composites. The melting temperature (T_m_) and crystallization temperature (T_c_) of PA10T increased with the incorporation of GNPs by the BMM process. The T_m_ of the composites was almost constant and slightly higher than that of neat PA10T, which was probably because the well-dispersed GNPs hindered the mobility of PA10T chains in the nanocomposites. The T_c_ of all the composites was higher than that of neat PA10T by about 5 °C, and could also be ascribed to the nucleation effect of the GNPs in the matrix. The increase in T_c_ with increasing GNP content was associated with providing more nucleation sites, which in turn reflected the good dispersity of the GNPs in PA10T [37,38]. What is more, the first melting peaks were smaller for all composites at T_m1_, which was due to the heterogeneous nucleation effect of GNPs, which bring about the formation of smaller and less perfect crystals. The melting enthalpy (ΔH_m_) for the composites was reduced compared to that of the PA10T when the GNP content was more than 7.5 wt%, which might have been due to fillers hindering the mobility of the polymer chains, thus retarding the nucleating process. The analysis also indicated that adding fillers to pure PA10T did not affect the crystallization process significantly.

Thermal stability is another essential property of thermal conductivity materials, with a decisive influence on the processing and applications of thermal conductive materials. The initial thermal decomposition temperatures (corresponding to the decomposition of 5 wt%) of the composites of BMM-2.5 wt%, BMM-5.0 wt%, BMM-7.5 wt%. and BMM-10.0 wt% were 422.7, 423.1, 421.1 and 423.1 °C, respectively, which were slightly higher than that of pure PA10T (421.6 °C). The values of T_d,10%_ and T_d,max_ were also improved when GNPs were added to the PA10T matrix. The reason for this is that the stacking crystalline graphite nanoplatelets on the surface of PA10T formed a barrier effect and hindered the movement of the molecular chains, which improved the resistance to thermal degradation and impeded the diffusion of depolymerization products from the polymer into the gas phase [39,40]. In the temperature range between 400 °C and 500 °C, the barrier effect of the graphite was destroyed. The weight loss of the samples were attributed to the degradation of PA10T resin. While heated above 500 °C, almost all PA10T components had been thermally decomposed and only graphite was left, which could then be used to characterize the content of GNPs. It was observed, from the residue of TGA testing at 600 °C. that the residue increased with the increase in GNP contents. In addition to the barrier effect of GNPs, the heat diffusion is likely also to have been accelerated by the GNPs due to their excellent thermal conductivity, which tends to reduce the decomposition temperature of composites. In summary, the barrier effect of two-dimensional flake filler GNPs plays a dominant role during thermal decomposition; thus, the thermal stability of composites is improved due to the introduction of GNPs.

### 3.4. DMA Analysis

**Figure 7:**
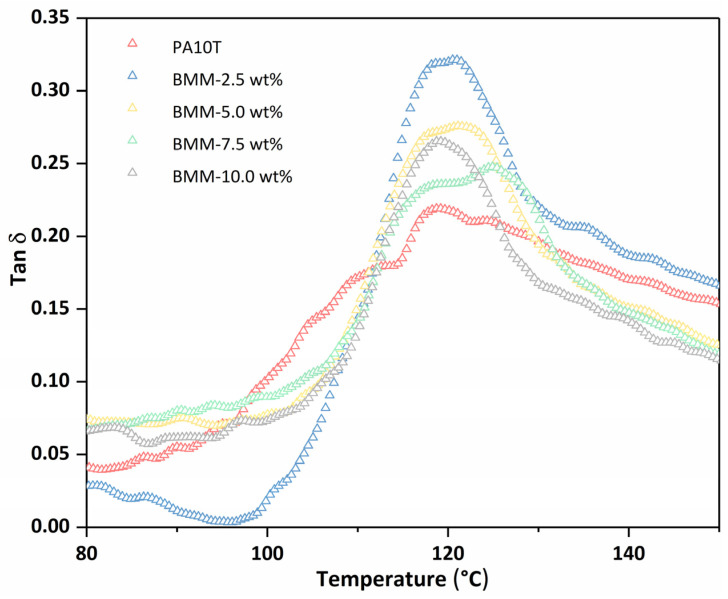
Dynamic damping factor (tan δ) of PA10T/GNP BMM samples.

Figure 7 shows the DMA curves of the neat PA10T and the PA10T/GNP BMM samples; Table 2 summarizes the T_g_, tan δ (the ratio of dissipated to stored energy) and storage modulus of the composites obtained from the curves. The T_g_ of the samples increased from 119.2 °C to 124.9 °C and then decreased to 119.2 °C, showing an inflection point located at approximately 7.5 wt% when the GNPs were filled from 0.0 wt% to 10.0 wt%. The changes in T_g_ were in agreement with the results of the crystallization study. The higher crystallinity and numerous small crystals in the nanocomposites with GNP contents of 2.5, 5.0 and 7.5 wt% provided greater restriction against the segmental motion of the amorphous chains; therefore, the T_g_ improved with the increase in GNP content (0.0 wt% to 7.5 wt%). However, when the GNP content was above 7.5 wt%, the fillers tended to be seriously aggregated, which resulted an the increase in the free volume among the polymer chains and a decrease in the available interface, nucleation sites, and crystallinity [41,42]; thus, the T_g_ of the composites decreased accordingly.

As shown in Table 2, the GNP-filled PA10T composites exhibited obviously higher tan δ peak height, since the introduction of fillers considerably increased the viscoelastic damping factor of the PA10T matrix. An increase in the tan δ value at the glass transition temperature indicated that the energy damping ability was increased. Storage modulus is a measure of stiffness of a polymer composite. The storage modulus of the composites at temperatures below and above T_g_ are also presented in Table 2. It is noted that the addition of GNPs had a significant effect on the storage modulus. The significant increase in the storage modulus below and above T_g_ of the composites is ascribed to the high stiffness behavior of the GNP filler, which can effectively constrain movement of the PA10T molecular chains in the vicinity of the nano reinforcements caused by the well-dispersed filler.

### 3.5. Thermal Conductivity

**Figure 8:**
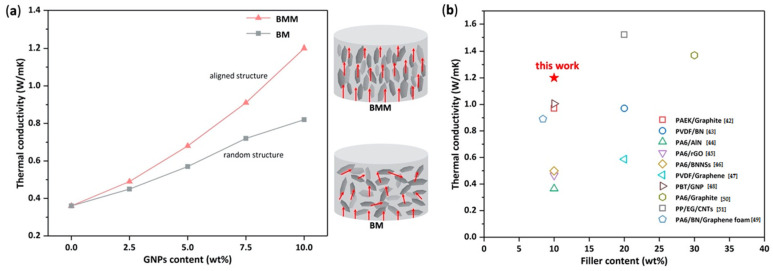
(**a**) Thermal conductivity of PA10T/GNP nanocomposites prepared by the BMM and BM processes; and (**b**) comparison with the values of thermal conductivity reported in previous studies.

The neat PA10T had a thermal conductivity of 0.36 W/(m·K), which was very low. The low thermal conductivity, which greatly restricts their practical applications, was similar to that of other polymers. When GNPs were added to the PA10T, the thermal conductivities of the composites increased with the increase in GNP content. The BMM at 2.5, 5.0, 7.5, and 10.0 wt% exhibited thermal conductivities of 0.49, 0.68, 0.91, and 1.20 W/(m·K), respectively, a remarkable enhancement compared with pure PA10T. In order to better explain the effects of GNPs and the BMM process on the thermal conductivity of the composites, composites prepared by the BM process were also compared in the experiment. As shown in Figure 8a, the BM sample also showed an improvement in thermal conductivity; however, the thermal conductivity of the BM sample was obviously lower than that of the BMM sample with the same GNP content. Because GNP has good thermal conductivity, its uniform dispersion in the PA10T matrix significantly improved the thermal conductivity of both the BMM and BM samples. Further, we propose that the remarkable improvement in the thermal conductivity of the BMM samples was probably ascribed to the following aspects. First, the large size of the obtained GNPs, as confirmed by AFM and the Raman spectrum, can rapidly conduct heat when heat is conducted to it. Second, the BMM process help to homogeneously disperse the GNPs in the PA10T matrix without obvious aggregation—as confirmed by the micro-X-ray CT—and then form an aligned GNP structure after melting extrusion, which is favorable for the formation of high-efficiency thermal conductive pathways for heat transfer [43,44]. In addition, the high interfacial affinity between PA10T and GNPs due to the π–π stacking interactions of phenyl groups in PA10T with aromatic structures on the GNP surfaces helps to avoid unwanted phonon scattering at the interfaces [45]. Figure 8b summarizes previous studies on thermal conductivity for thermoplastic polymer composites with thermal conductive fillers [46,47,48,49,50,51,52,53,54,55]. The data show that the BMM process exhibits a better ability to enhance thermal conductivity among the reported composites at a low filler content. More importantly, the BMM process is a green and economic process, as the BMM composites were prepared through solid-state ball milling and melt-blending without the use of solvents, resulting in good processability and decreased production costs. Based on these results, we believe that the BMM process is advantageous in preparing well-dispersed and aligned nanoplatelet-filled composites, compared to other methods.

### 3.6. Mechanical Properties

**Figure 9:**
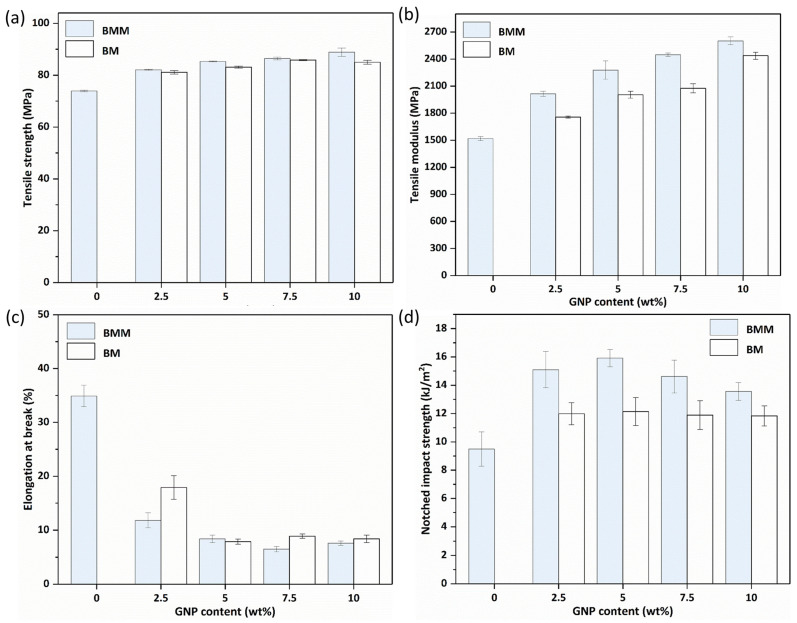
Mechanical properties of the PA10T/GNP nanocomposites prepared by BMM and BM processes: (**a**) tensile strength; (**b**) tensile modulus; (**c**) elongation at break; and (**d**) notched impact strength.

According to tensile tests in Figure 9, the addition of GNPs brought an obvious increase in tensile strength, tensile modulus, and impact strength of both the BMM and BM samples compared with the PA10T matrix, as well as a decrease in the elongation at break. This indicated that restraining the movement of polymer molecular chains and bearing the external forces greatly contributed to the reinforcement effect of rigid filler on PA10T matrix [56,57] It is worth noting that the large decrease in the elongation at beak is a common problem encountered in rigid-filler-filled polymer composites.

For the BM-10.0 wt% sample, in Figure 9a–c, tensile strength and strength modulus improved from 73.8 MPa to 85 MPa and 1519.1 MPa to 2437.3 MPa, respectively, but elongation at break was decreased to 8%, owing to structural defects caused by the graphite nanoplatelets. The impact strength of the nanocomposites increased from 9.5 kJ/m^2^ to 11.8 kJ/m^2^, as shown in Figure 9d. A comparison of the mechanical properties of the two samples indicated that both the BMM and BM composites showed similar tendencies in mechanical properties as the GNP content increased. However, the tensile strength, tensile modulus, and impact strength values of the BMM samples were 88.9 MPa, 2601.6 MPa and 13.6 kJ/m^2^ at 10 wt% of the loaded GNP, which were obviously higher than those of the BM samples at the same filler loading percentage. The more uniform dispersion of the GNPs in the BMM samples was an important factor in composite reinforcement, as the load can effectively transfer from the matrix to the filler and exhaust energy when the composites are distorted, or under external force. In addition to dispersion, filler alignment is another important factor for fabricating high-performance nanocomposites. For the BMM process, GNPs were preferentially aligned parallel to the flow direction of the PA10T matrix during melt-blending, which achieved alignment to a certain extent, reduced agglomeration, and improved the dispersion of the filler in the matrix, as shown in the micro-CT images. The aligned nano-fillers in the composites led to higher reinforcement efficiency, which enabled the composites to bear greater loads along the orientation direction during tensile testing. It should be noted that there may be a high affinity between PA10T and GNPs due to the π–π stacking interactions of phenyl groups in PA10T with the aromatic structures on the GNP surfaces, which can bring about strong interface adhesion for effective stress transfer under loading. Therefore, the results confirm the superiority of the BMM process proposed in this work.

## 4. Conclusions

In summary, we have proposed a novel fabrication method that combines the benefits of both ball milling and melt-blending to obtain highly thermally conductive and mechanically PA10T/graphite nanoplatelet (GNP) composites. The exfoliation of the graphite via ball milling was characterized by Raman spectra and AFM. The dispersion of the GNPs is demonstrated by SEM observation and X-ray micro-CT. The thermal conductivity coefficient of composites was markedly improved, at 1.20 W/(m·K) with 10 wt% GNPs, owing to the well-dispersed and aligned distribution of GNPs, which facilitate the formation of effective thermal conductive pathways. In addition, the tensile strength, impact strength and tensile modulus of composite increased from 73.8 MPa to 88.9 MPa, 9.5 kJ/m^2^ to 13.6 kJ/m^2^, and 1519.1 MPa to 2601.6 MPa at a GNP filler loading of 10 wt%. These excellent comprehensive performances make PA10T/GNP composites with aligned structures a promising candidate material for thermal management applications.

## Figures and Tables

**Table 1:** DSC and TGA data of the PA10T/GNP samples.

Samples	T_m_ (°C)	T_c_ (°C)	ΔH_m_ (J/g)	T_d,5%_ (°C)	T_d,10%_ (°C)	T_d,max_ (°C)
PA10T	314.5	288.9	59.2	421.6	435	464.6
BMM-2.5 wt%	314.9	293.8	61.2	422.7	436.5	467.3
BMM-5.0 wt%	314.7	294.1	60.7	423.1	435.8	466.1
BMM-7.5 wt%	315.6	293.8	58.7	421.1	435.1	466.2
BMM-10.0 wt%	314.9	292.8	56.6	423.1	437.6	467.2

**Table 2:** T_g_, tan δ and storage modulus of the PA10T/GNP BMM samples.

Samples	T_m_ (°C)	T_c_ (°C)	ΔH_m_ (J/g)	T_d,5%_ (°C)	T_d,10%_ (°C)	T_d,max_ (°C)
PA10T	314.5	288.9	59.2	421.6	435	464.6
BMM-2.5 wt%	314.9	293.8	61.2	422.7	436.5	467.3
BMM-5.0 wt%	314.7	294.1	60.7	423.1	435.8	466.1
BMM-7.5 wt%	315.6	293.8	58.7	421.1	435.1	466.2
BMM-10.0 wt%	314.9	292.8	56.6	423.1	437.6	467.2

## Data Availability

Data are contained within the article and Supplementary Materials.

## Supplementary Materials

The following supporting information can be downloaded at: https://www.mdpi.com/article/10.3390/molecules29133141/s1. The synthesis of PA10T; the characterization methods.
