# Supplementary material for: Easy and Green Method to Fabricate Highly Thermally Conductive Poly(decamethylene terephthalamide)/Graphite Nanoplatelets Nanocomposite with Aligned Structure
Source: Molecules. 2024 Jul 2;29(13):3141. doi: 10.3390/molecules29133141 (PMC11243090; doi:10.3390/molecules29133141)
Supplement: Supplementary file 1 [file molecules-29-03141-s001.zip › molecules-3070204-supplementary.pdf]

Supporting Information

Easy and Green Method to Fabricate Highly Thermally Conductive Poly(decamethylene terephthalamide)/Graphite Nanoplatelets Nanocomposite with Aligned Structure

Pengyuan Xu [1], Tianhao Ai [1,*], Pingli Wang [1,2,*] and Junhui Ji [1,2]

[1] National Engineering Research Center of Engineering Plastics and Ecological Plastics, Technical Institute of Physics and Chemistry, Chinese Academy of Sciences, Beijing 100190, China; xupengyuan19@mails.ucas.ac.cn (P.X.); jhji@mail.ipc.ac.cn (J.J.)

[2] Hainan Degradable Plastics Technology Innovation Center, Haikou 571137, China

* Correspondence: aitianhao17@mails.ucas.ac.cn (T.A.); pingliwang@mail.ipc.ac.cn (P.W.)

Experimental

Materials

Bio-based 1,10-diaminodecane (DMD) was purchased from Wuxi Yinda Nylon Co., Ltd. (Wuxi, China). Commercially available Terephthalic acid (TPA) was purchased from China Petrochemical Group Co., Ltd. Benzoic acid (BA) and sodium hypophosphite (SHP) were purchased from XiLong Chemical Co., Ltd. (Guangzhou, China). Concentrated sulfuric acid (96%) was purchased from Beijing Chemical Reagent Company. High-purity nitrogen (N_2, 99.999%) was purchased from the Beijing Huanyu Jinghui Gas Technology Co., Ltd. Deionized water was self-made. All reagents were used as received.

Synthesis of PA10T

The procedures listed below were as follows: (a) DMD (1.01 mol), TPA (1 mol), ND aqueous suspension, BA (0.01 mol), and SHP (0.1 wt%, based on the total weight of monomers) were added into a stirred autoclave. Then, the stirring apparatus was set to 50 rpm and the autoclave was purged with N_2 for air replacement three times. The autoclave was heated to 80 °C and was held for about 1 h to obtain nylon salt. Subsequently, the autoclave was heated to 240 °C for 3 h and for about 1.5 h. The pressure of the autoclave was maintained within 2.5 MPa by discharging steam and the nylon salt was converted to a prepolymer under high-temperature and high-pressure conditions. After that, the molecular weight of the prepolymer gradually increased with the discharge of water vapor, and the pressure was steadily decreased to atmospheric

pressure within 1 h and cooled down to room temperature to obtain PA10T prepolymers. After pulverization, the prepolymers were put into a vacuum oven. The vacuum oven was evacuated within 20 Pa, heated to 250 °C, and held for 5 h. Finally, the vacuum oven was naturally cooled down to room temperature after the polymerization was completed.

Characterization

Intrinsic viscosity and molecular weight of PA10T

Intrinsic viscosity measurements were performed with 0.005 g/mL polymer solution (96% concentrated sulfuric acid) using an Ubbelohde viscometer in a water bath at 25.00 ± 0.05 °C. The efflux time of concentrated sulfuric acid and the polymer solution were recorded as t_0 and t_1, respectively. The intrinsic viscosity, recorded as [η], was calculated by the Solomon and Ciuta relationship as follows:

$$[\eta]=\frac{\sqrt{2(\eta_{sp}-\ln\eta_r)}}{c}$$

where c (concentration of the polymer solution) is 0.005 g/mL, η_{sp} (specific viscosity) was calculated by ($t_1/t_0 - 1$), and η_r (relative viscosity) was obtained by t_1/t_0. The Mark–Houwink equation was used to estimate the M_w of the PA10T and 10T/ND nanocomposites:

$$[\eta] = 0.0003824(\mathrm{dL/g})M_w^{0.8159}$$

	[η] (dL/g)	M_w (g/mol)

PA10T	0.93	14100
